# Impact of Collagen on the Rheological and Transport Properties of Agarose Hydrogels

**DOI:** 10.3390/gels11060396

**Published:** 2025-05-27

**Authors:** Veronika Richterová, Alžběta Gjevik, Ondřej Vaculík, Jakub Vejrosta, Miloslav Pekař

**Affiliations:** 1 Institute of Physical and Applied Chemistry, Faculty of Chemistry, Brno University of Technology, 61200 Brno, Czech Republic; 2 Institute of Scientific Instruments of the Czech Academy of Sciences, 61200 Brno, Czech Republic

**Keywords:** agarose, collagen, hydrogels, rheology, mesh size, transport, diffusion coefficient

## Abstract

This work investigated how collagen addition affects the rheological and transport properties of agarose hydrogels. Collagen did not affect the rheological character of hydrogels (i.e., the overall shape of amplitude and frequency response curves) but changed their viscoelastic moduli and mesh size dependent on the concentration of both constituents. The diffusion coefficients of the oppositely charged model dyes eosin B and methylene blue were determined in all hydrogels and demonstrated a profound effect of electrostatic interactions. Comparison with similar work with fibroin addition showed that while the effects of these proteins on the viscoelastic properties of a polysaccharide network can be similar, their impact on network transport properties may be different.

## 1. Introduction

The use of hydrogels, three-dimensional polymer materials with unique properties and a considerable ability to absorb and retain water [1], has expanded greatly since their first application as contact lenses [2]. In particular, hydrogels have proven to be versatile materials for creating environments mimicking the natural extracellular matrix (ECM) [3,4]. However, the ECM is not just a gel-like bioenvironment, but also a hydrocolloid system encompassing a rich array of molecules that collectively shape the biological, chemical, and mechanical attributes of this extrinsic cellular medium [5,6]. Nevertheless, the biocompatibility, biofunctionality, porosity, and viscoelasticity [7,8] of hydrogels and the variety of polymer types available allow the creation of hydrogels with unique and specific properties suitable for the desired applications [9].

ECM incorporates various fibers such as collagen or elastin, which inherently contribute to the conducive cellular milieu. These fibrous molecular frameworks serve as pivotal structural elements, exerting influence not only over the mechanical characteristics of the matrix but also actively participating in shaping its biochemical and biological properties [10,11]. Despite this complex composition, model studies on the influence of isolated components are meaningful and called for [12]. A hydrogel matrix into which fibrous structures are incorporated can thus be considered as a primitive, basic model for elucidating the effects of fibers on the properties of the hydrogel network.

One suitable candidate for creating a hydrogel network is agarose. This naturally occurring linear polysaccharide, derived from selected seaweed species within the agar genus, exhibits promising attributes for biomedical applications [13]. Thanks to its excellent water retention, biocompatibility, and adjustable nutrient permeability, agarose is widely utilized in medical applications, particularly for controlled drug release and cell encapsulation. It is also used as a wound dressing and a bioink; in the development of artificial corneas, skin, and cartilage; and as an ingredient in the food industry [14,15,16,17]. While pure agarose hydrogels offer many advantages, they are, unfortunately, not biodegradable in mammals, and their mechanical properties and porosity need to be carefully balanced to support cell growth and nutrient diffusion [18,19]. Their lack of structural adaptability may be compensated for by adding other types of polymers. Collagen is a structural protein, composed of amino acids bound together in a triple helix structure, that provides strength, elasticity, and framing to various tissues across many different species [16,17,20]. Collagen, being the most abundant protein in the human body, is a biocompatible and biodegradable material with low immunogenicity, and its hydrogels are widely used in tissue engineering and regenerative medicine [21].

This work thus used agarose as the biopolymer forming a hydrogel network and collagen as the fibers embedded in this hydrogel matrix. Its aim was to investigate the effect of the fibrous component on the transport and mechanical properties of the hydrogel matrix. Specifically, the diffusion of model dyes in agarose–collagen systems and these systems’ oscillatory rheology were studied. This work is intended as a response to the call for studies on the behavior of simple systems containing only a few components found in much more complex biological environments [12]. It can be further noted that it has already been proven that the combination of agarose and collagen is beneficial for increasing cell adhesion, biofunctionality, cell migration, and more [22,23].

## 2. Results and Discussion

### 2.1. Oscillation Rheometry of Agarose–Collagen Hydrogels

The results from the amplitude sweep tests are summarized in Table 1 (and the data obtained from the measurements are listed in the Supplementary Materials—Figures S1–S3).

In the case of pure agarose hydrogels, the expected behavior occurred; that is, with an increase in agarose concentration, the hydrogels became mechanically more resistant (stiffer), which was reflected in the values of both viscoelastic moduli. As the concentration of agarose increased, the LVER also decreased, i.e., the gels were less resistant to increasing mechanical stress, and, at the same time, the crossover points shifted to lower values of the deformation amplitudes.

**Figure 1:**
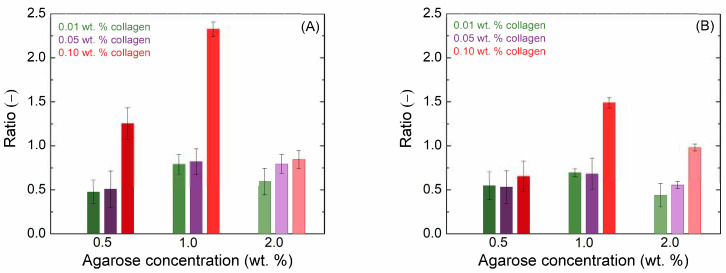
Ratio of the amplitude sweep (**A**) and frequency sweep (**B**) viscoelastic modulus *G*′ of the gel with the addition of collagen at various concentrations to that of the pure agarose gel.

Different additions of collagen have different effects depending on the concentration of agarose. Due to the influence of collagen, the values of the viscoelastic modules first decreased, but as the concentration increased, the hydrogel network gradually became stronger. The increased concentration of agarose shortened the LVER; its length differed very little with different additions of collagen. The influence of collagen on the crossover point was more pronounced. In all cases with the addition of collagen, there was first a shift to higher strain amplitude values at the crossover point; but, with high collagen content, the crossover points returned to almost the original values of the reference hydrogel. Figure 1A shows the ratio of the elastic moduli of the pure agarose gel to the moduli of the agarose gel with the addition of collagen at different concentrations. It can be observed that the greatest influence of collagen was observed for 0.5 wt. % agarose with lower collagen concentrations and for 1.0 wt. % agarose with the highest collagen addition, while the influence on 2.0 wt. % agarose was minimal.

Frequency sweeps confirmed a very weak dependency of both moduli on frequency (Figures S1–S3), known for (pure) agarose hydrogels [24]. This is typical for solid-like materials [25]. Furthermore, the storage modulus is always and significantly higher than the loss modulus, confirming the dominancy of the elastic response of all of the investigated hydrogels. Collagen addition did not change the rheological character of agarose hydrogels; it affected the values of their rheological moduli. Figure 1B shows the same ratio as Figure 1A but determined from frequency sweep measurements at 5 rad·s^−1^. The observed trends are similar to those in Figure 1A.

Similar trends were also observed for the loss modulus. The rheological results suggest that the impact of collagen on the viscoelastic properties of agarose hydrogels is most pronounced at lower agarose concentrations and higher collagen additions, while minimal effects are observed at higher agarose concentrations.

**Figure 2:**
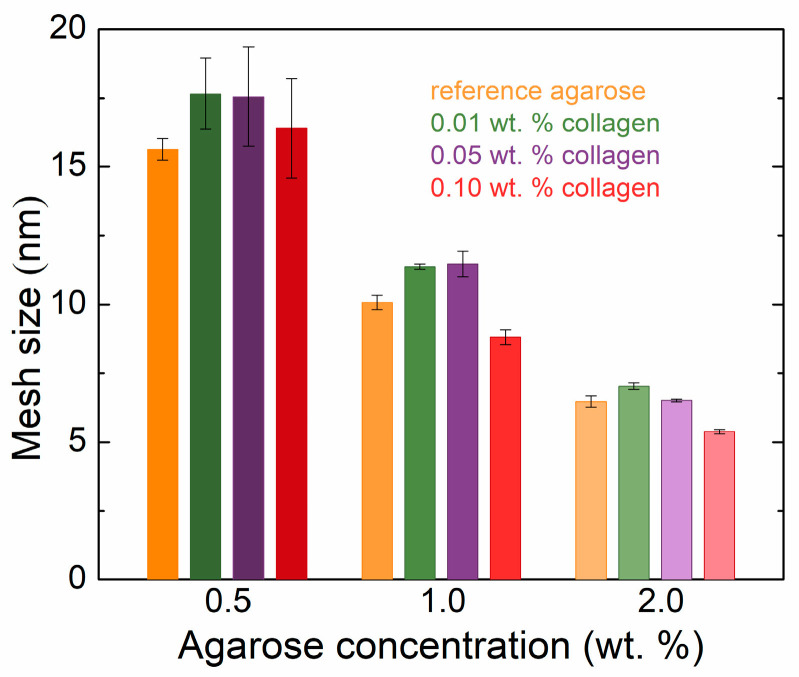
Rheologically calculated mesh sizes of agarose hydrogels with the addition of collagen in different concentrations.

The presence of collagen in the agarose samples also affects the resulting mesh sizes in the hydrogel network calculated from the rheometry data (Figure 2). Increasing the concentration of agarose resulted in decreasing mesh sizes regardless of the presence of collagen. Samples with the same concentration of agarose but with different collagen contents first showed an increase in the size of the meshes (i.e., at the lowest collagen concentration), but as the concentration of collagen increased, this mesh size gradually decreased. At higher concentrations of agarose, with higher additions of collagen, the mesh sizes were smaller than in the reference (pure) hydrogel. At lower concentrations, collagen appeared to interfere with the formation of the agarose network, leading to an increase in mesh size. In contrast, at higher concentrations, collagen likely acted as a filling component, reducing the mesh size by occupying voids within the hydrogel structure.

In a recent study [26], we studied the effect of silk fibroin, another protein which is, however, not found in mammals. A comparison of selected physicochemical properties of collagen and silk fibroin is presented in Table S1 [27,28,29,30,31,32,33,34,35]. It should be stressed that fibroin was added in higher amounts, viz. from 0.6 to 4.5 wt. %. Nevertheless, the effect of fibroin on the rheological moduli in the linear viscoelastic region as well as on the moduli of the plateau in the frequency sweep test was qualitatively similar to the effect of collagen observed in this work. Some difference was observed only for the 1 wt. % agarose hydrogels with the first two protein additions. In the case of fibroin, a slight increase in the storage moduli was observed. The mesh sizes of the agarose hydrogels were also very similar for both fibroin and collagen additions. The trends with added collagen and fibroin are nearly identical, with only a slight difference in the 1 wt. % agarose hydrogels.

### 2.2. Transport Properties of Model Dyes in Agarose–Collagen Hydrogels

**Figure 3:**
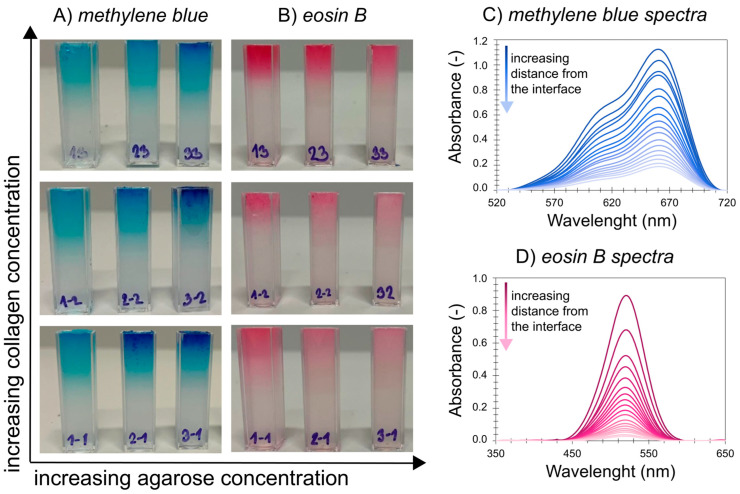
Different progress of transport of (**A**) methylene blue and (**B**) eosin B through agarose–collagen hydrogels after 72 h of immersion in the corresponding dye solution and examples of collected spectra for (**C**) methylene blue and (**D**) eosin B in 0.5 wt. % agarose hydrogel with 0.01 wt. % collagen.

As can be observed in Figure 3, the dye passage rate and interface concentration exhibit different behavior in the shown diffusion media, and both agarose concentration and collagen concentration significantly affect dye transport. The obtained concentration profiles of both dyes are presented in the Supplementary Materials (Figures S4 and S5). The values of the effective diffusion coefficients and the theoretical concentrations at the hydrogel interface of both methylene blue and eosin B are summarized in Table S2. The diffusion data could be fitted very well by Equation (3) regardless of the potential obstruction by, and electrostatic effects of, the added protein. These effects thus did not substantially change the transport mechanism at the used composition. Nevertheless, we call the determined diffusion coefficient effective because it can still include more than just pure Fickian effects.

**Figure 4:**
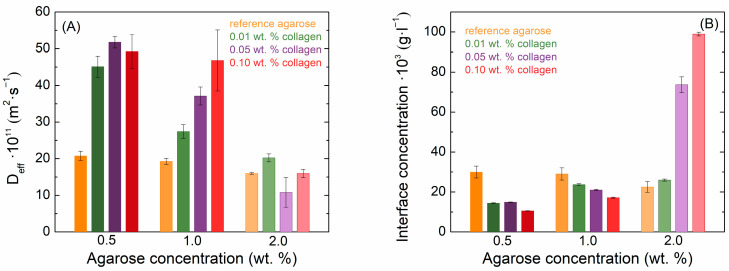
Values of effective diffusion coefficients (**A**) and theoretical interface concentrations (**B**) of methylene blue in agarose–collagen hydrogels.

As the agarose concentration increased, the diffusion velocity of methylene blue slowed down, and the theoretical concentration at the cuvette’s interface slightly decreased due to the denser hydrogel network. The effect of collagen on the diffusion coefficient is shown in Figure 4A, and on the theoretical concentration at the interface in Figure 4B. From the values of the determined diffusion coefficients, it can be seen that the addition of collagen significantly increased the speed of methylene blue transport in samples with a lower concentration of agarose (0.5 and 1.0 wt. %). In the case of 2.0 wt. % of agarose, collagen no longer had a profound or systematic effect and affected the diffusion coefficient only slightly. The addition of collagen resulted in a reduction in the methylene blue concentration at the interface across all samples, with the exception of 2% agarose. Likewise, increasing collagen concentrations further decreased the interface concentration, though in the case of 2% agarose, the concentration increased instead.

**Figure 5:**
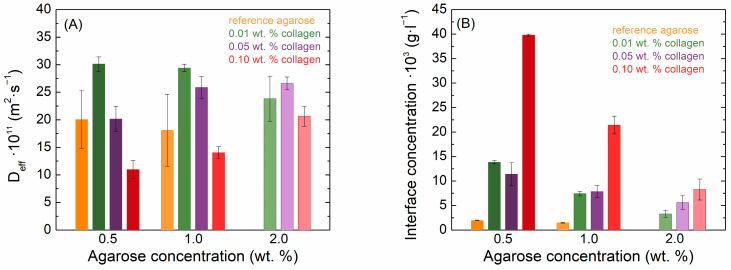
Values of effective diffusion coefficients (**A**) and theoretical interface concentrations (**B**) of eosin B in agarose–collagen hydrogels.

Also, the diffusion coefficient and the concentration at the interface of eosin B decreased with increasing agarose concentration in pure hydrogels (Figure 5). However, the transport of eosin B in agarose–collagen hydrogels showed a different behavior from methylene blue. With the addition of collagen, there was an increase in the absorbance of eosin B in the samples. While the movement in 2.0 wt. % agarose could not be determined due to the high turbidity and low absorbance of eosin B, samples with collagen manifested a higher absorbance and it was possible to obtain concentration profiles in them. As with methylene blue, the presence of collagen at the lowest concentration increased the values of the effective diffusion coefficients in 0.5 and 1.0 wt. % agarose. However, a further increase in collagen concentration suppressed eosin B diffusion, and at its highest concentration the effective diffusion coefficient was even smaller than in the reference agarose hydrogel (Figure 5A). In contrast to methylene blue, the concentration of eosin B at the interface increased upon the addition of collagen in these samples, particularly for the highest collagen concentration (Figure 5B). In 2.0 wt. % agarose, it is reasonable to expect a somewhat lower diffusion coefficient than at lower concentrations; in these cases, the addition of collagen also increased the diffusion coefficient, but the collagen concentration had only a minor effect, similar to the case of methylene blue. Also, the interfacial concentration of eosin B was increased when collagen was added.

When comparing the rheological and transport properties of the prepared hydrogels, it is evident that the observed changes in dye diffusion cannot be explained by mechanical parameters alone. Increasing the agarose concentration resulted in a denser network, reflected in both reduced mesh size and lower diffusion coefficients for both dyes, which is in line with expectations. In the case of collagen addition, however, the relationship is less direct. Rheological data showed non-linear effects on the viscoelastic moduli and mesh size depending on both agarose and collagen content. For example, at lower agarose concentrations (0.5 and 1.0 wt. %), low collagen content led to increased mesh size and accelerated methylene blue transport. At a higher collagen content, the mesh size decreased again, yet diffusion remained enhanced. These trends suggest that electrostatic interactions between the dyes and the matrix, particularly the surface charge behavior of collagen, also play a significant role. The overall transport response thus likely results from a combination of structural changes (mesh size) and physicochemical interactions (charge), whose relative contributions vary with composition. This indicates that rheological and transport results are connected through the interplay of network architecture and dye–matrix interactions, and that neither set of data alone fully explains the observed behavior.

The mesh size values derived from rheological data provide further insight into the steric component of molecular transport. Larger molecules such as eosin B are more susceptible to obstruction in denser networks, while smaller molecules like methylene blue can still diffuse more freely even as the mesh size decreases. This is supported by the observation that the decline in diffusion with increasing agarose concentration is more pronounced for eosin B. The effect of collagen is particularly interesting, as it temporarily loosens the network structure at low concentrations, which facilitates transport, but at higher concentrations the denser composite network suppresses diffusion again, especially for the larger dye.

These findings support the view that mesh size and steric accessibility alone do not fully govern the transport behavior. Methylene blue, a cationic dye, likely forms weak electrostatic interactions with negatively charged residues on collagen, which may contribute to its continued mobility even as the gel becomes denser. In contrast, the anionic eosin B may be partially excluded from the collagen-rich matrix due to electrostatic repulsion, resulting in lower partitioning and reduced effective diffusion. In addition, collagen incorporation increases tortuosity and structural heterogeneity, both of which are known to hinder transport by extending diffusion pathways and introducing physical obstructions. These combined effects likely underlie the non-monotonic diffusion trends observed with increasing collagen concentration, where initial network loosening facilitates transport, but higher protein levels reduce mobility due to obstruction and possible binding.

When compared with the effect of silk fibroin on the transport properties of hydrogels [26], the effect of collagen on the transport properties of the gels in this study was different, unlike in the case of rheological properties. The addition of collagen accelerated the diffusion of methylene blue; this effect was strongest for 0.5 and 1 wt. % agarose hydrogels and also increased with increasing collagen concentration. In the case of fibroin, increasing its concentration decreased the methylene blue diffusion coefficient, with the highest fibroin concentration even resulting in a value below that determined for the pure agarose hydrogel. The theoretical interface concentration of methylene blue decreased with increasing collagen concentration for the 0.5 and 1 wt. % agarose hydrogels and was also lower than in the case of the pure hydrogel. In contrast, this concentration increased for 2 wt. % agarose hydrogels with collagen. In the case of fibroin, the increased interface concentration, higher than for pure hydrogel, was found only for the highest fibroin concentration in 0.5 and 1 wt. % agarose hydrogels. Silk fibroin decreased the diffusion coefficient of eosin B in all agarose hydrogels, in contrast to collagen addition, where only the highest collagen concentration used resulted in an observed decrease below the value for pure hydrogel. In contrast, both proteins significantly increased the interface concentration of eosin B. Silk fibroin, with a zeta potential of –12 mV, is weakly negatively charged [26], while collagen is more strongly negatively charged at physiological pH [36]. The observed differences in diffusion coefficients and interfacial concentrations of eosin B and methylene blue at high agarose concentrations can be attributed primarily to charge interactions between the dyes and the proteins, with structural differences between fibroin and collagen playing a secondary role. Both proteins may incorporate differently into the denser hydrogel network, potentially influencing transport properties; however, the overall impact on diffusion coefficients remains relatively minor. Notably, both dyes exhibit a tendency to accumulate at the interface, reinforcing the idea that electrostatic interactions are the dominant factor governing their transport behavior. The larger molecular structure of collagen compared to fibroin may further contribute to increased steric hindrance within the hydrogel network, modulating diffusion dynamics.

While UV–VIS spectroscopy provides robust spatially resolved concentration profiles and is widely used for studying diffusion in agarose- and collagen-based hydrogels [37,38], it does not directly differentiate between free and bound dye fractions or local structural variations. As the method yields effective diffusion coefficients that reflect the overall transport behavior under given conditions, it is particularly suitable for comparative analysis across different hydrogel compositions. For more detailed insight into binding dynamics or microstructural heterogeneity, advanced techniques such as fluorescence-based methods or NMR have been applied in previous studies [39,40,41]. These techniques could be explored in future work to gain additional insight into binding dynamics and local mobility within composite hydrogels.

The trends observed in our study are in agreement with previous reports on dye diffusion in agarose- and collagen-based hydrogels. Similar non-linear effects of protein addition on molecular transport have been described in composite systems, where charged macromolecules such as gelatin introduced both binding sites and structural obstacles for diffusing species [42]. Moreover, selective interactions between cationic dyes and negatively charged networks have been shown to modulate effective diffusion coefficients and concentration profiles [43]. Our findings therefore support the broader understanding that molecular transport in biomimetic hydrogels is governed by a combination of mesh architecture, electrostatic interactions, and charge-selective retention.

Agarose–collagen hydrogels have been widely studied for tissue engineering due to their mechanical tunability and biofunctionality. Previous studies showed that collagen addition supports chondrocyte and nucleus pulposus cell adhesion, viability, and mechanotransduction, while maintaining the overall hydrogel structure [23,44]. However, these works primarily focus on the biological outcomes without detailing the underlying physicochemical mechanisms. Our study complements these findings by systematically analyzing how collagen alters the viscoelasticity and diffusion of small molecules in agarose hydrogels. These insights are essential for designing cell-supportive matrices with controlled mechanical and transport properties.

## 3. Conclusions

The presence of collagen did not affect the overall viscoelastic properties of the investigated hydrogels (the shapes of curves describing the strain amplitude and the frequency dependence of the viscoelastic moduli remained unchanged). However, adding collagen affected the values of both the storage and loss moduli as well as the mesh sizes calculated from the frequency sweep tests. With an increasing concentration of collagen, the moduli could be either higher or lower than those of the original hydrogel depending on the concentration of both constituents. A similar pattern was observed concerning mesh sizes; at lower concentrations, collagen likely interfered with the formation of the agarose networks, while at higher concentrations, it also acted as a filler.

The effect of collagen on the diffusion of two oppositely charged dyes was more profound and showed that electrostatic interactions play an important role. Comparison with a similar study on fibroin addition showed that while the effects of these proteins on the viscoelastic properties of a polysaccharide network can be similar, their impact on network transport properties may be different. Fibrous protein structures affect network properties by their incorporation into the network architecture and by introducing sites for electrostatic interactions.

## 4. Materials and Methods

### 4.1. Chemicals

Agarose (routine Agarose E for molecular biology, Condalab, Madrid, Spain), eosin B, and methylene blue (Sigma-Aldrich, Prague, Czech Republic) were used without further purification. Collagen hydrogel mass (VUP Medical, Brno, Czech Republic; 2.8 wt. %) was dispersed with deionized water to prepare the diluted collagen suspension (0.1 wt. %) used for sample preparation. This mass is a commercial product approved for medical applications and used mainly for the fabrication of artificial blood vessels; therefore, it was selected as a collagen source. It is isolated from bovine skin.

The collagen hydrogel mass was characterized by several techniques and the results are shown in Figure S6. Infrared spectroscopy (FT-IR spectrometer NICOLET IS 50, Thermo Fisher Scientific, Waltham, MA, USA) was used to characterize the structure of collagen with the following parameters: ATR mode, 32 scans, and a resolution of 4 cm^−1^. Differential scanning calorimetry (DSC analyzer Q2000, TA Instruments, New Castle, DE, USA) and dynamic mechanical analysis using a rheometer (Discovery HR-2, TA Instruments, New Castle, DE, USA) were used to analyze the thermal properties of collagen. DSC measurements were performed using an aluminum pan with a heating rate of 2 °C/min, while DMA was conducted in a parallel plate geometry with a diameter of 20 mm. The pH of the collagen mass was measured using a pH meter (SevenEasy S20, Mettler Toledo, Columbus, OH, USA) and was determined as 3.383 ± 0.012.

### 4.2. Preparation of Hydrogels

A set of three reference agarose hydrogels (0.5 wt. %, 1 wt. %, and 2 wt. %) was prepared. Subsequently, sets of samples were prepared for each concentration of agarose with three different additions of collagen suspension. The final collagen concentrations were 0.01 wt. %, 0.05 wt. %, and 0.1 wt. %. The collagen concentration in native ECM is typically higher depending on the specific tissue. However, in hydrogel models, collagen is commonly used at lower concentrations to balance biomimetic properties, mechanical stability, and transport dynamics [45]. A thermoreversible gelation of the aqueous solution was used for the preparation of agarose hydrogels—the required amount of agarose powder was dissolved in deionized water at 80 °C, and subsequent cooling to room temperature led to the formation of hydrogels. Since collagen denatures at higher temperatures, agarose–collagen samples were prepared by mixing a collagen suspension with a nearly cooled, but not yet solidified, agarose solution, to obtain consistent mechanical properties across samples. This temperature was around 35 °C, while collagen denatures at around 37–40 °C (Figure S6), so the temperature was sufficient for collagen to retain its native properties [46,47]. Samples were transferred into PMMA spectrometric cuvettes (10 × 10 × 45 mm) for diffusion experiments, or into a Petri dish (25 mm diameter, 1 mm height) for rheology experiments. All samples were freshly prepared in triplicate, and the subsequent experiments were consistently repeated using freshly prepared samples to ensure the reliability and reproducibility of the results. In the graphs, repetitions are represented by error bars.

### 4.3. Oscillation Rheometry of Agarose–Collagen Hydrogels

The viscoelastic properties of the hydrogels were studied using oscillatory rheology measurements, which were taken in two steps. In general, amplitude tests, with a constant frequency of oscillations, first serve to determine the linear viscoelastic region (LVER), in which irreversible deformation of the sample does not occur. For frequency tests, the deformation amplitude value is then selected from this region. From the oscillatory rheology measurement, in addition to the LVER, the values of storage and loss moduli *G*′ and *G*″ can also be determined, which reflect the density of the crosslinking of hydrogels and the strength of the resulting hydrogel network. The crossover point of these moduli is the change from solid-like to liquid-like behavior of the hydrogel [48]. The crosslinking density $\rho_{x}$ (mol·m^−3^) can be calculated using the value of the elastic modulus on the plateau $G_{e}$ (Pa), the universal gas constant R (J·mol^−1^·K^−1^), and the thermodynamic temperature T (K) [49,50,51]:

(1) $$\rho_{x}=\frac{G_{e}}{RT}$$

The mesh size ξ (m) can be calculated using the crosslinking density and Avogadro’s number $N_{A}$ [52]:

(2) $$\xi =\sqrt[{3}]{\frac{6}{\pi \rho_{x}N_{A}}}$$

Rheology experiments were performed using an Anton Paar MCR72 rotational rheometer employing cross-hatched 25 mm parallel plate geometry. Each measurement was performed for at least two repetitions with a freshly prepared sample. A summary of parameters is presented in Table S3.

### 4.4. Transport Properties of Model Dyes in Agarose–Collagen Hydrogels

The diffusion from a constant source method was used to study the transport of methylene blue (positive charge) and eosin B (negative charge) in the prepared hydrogels. These dyes were selected for their suitability for the spectroscopic monitoring of transport, while also having a history of medical applications. Methylene blue has been used in the treatment of malaria and psychiatric disorders and exhibits cytoprotective effects [53,54,55]. Similarly, eosin B has been applied in malaria treatment, and as a staining agent in medical diagnostics [56,57]. The structural formulas and molecular weights of the dyes used are given in Table S4.

The non-stationary macroscopic observation of diffusion is based on the spectrophotometric monitoring of the dye in solution, in which the sample is immersed, at different distances from the hydrogel–solution interface at different time intervals.

The cuvette with the sample was immersed in an aqueous solution of dye under continuous stirring and covered with parafilm. UV–VIS spectra were collected using a Varian Cary 50 UV–VIS spectrophotometer equipped with a special accessory allowing spectra collection at different heights of the cuvette. At defined time intervals (24, 48, and 72 h for methylene blue; 72 and 196 h for eosin B), the cuvettes were taken out of the dye solution and the UV–VIS spectra were collected within the hydrogel at 1 mm intervals, starting from the hydrogel–solution interface and proceeding deeper into the hydrogel. Measurements continued until the dye absorbance could no longer be reliably detected due to either limited diffusion or increased turbidity. All parameters of diffusion experiments for both dyes are summarized in Table S5.

Dye concentrations from the absorption maxima were calculated on the basis of calibration samples with known concentrations of both dyes. According to the dependence of dye concentration on the distance from the hydrogel–solution interface, dye concentration profiles were created.

A mathematical formula originating from Fick’s law can be used to fit each concentration profile, and the effective diffusion coefficient $D_{eff}$ (m_2_·s^−1^) can be determined:

(3) $$c_{x}{=c}_{0}ERFC\frac{x}{\sqrt{4D_{eff}t}}$$

where $c_{x}$ and $c_{0}$ are the concentrations of dye in the hydrogel (g·L^−1^) at distance x (m) from the hydrogel–solution interface or at the interface, respectively, and t is the time of diffusion [58].

The concentration profiles were fitted by Equation (3) using the Solver tool in MS Excel, where one of the fitting parameters is the effective diffusion coefficient at a specific time. The final effective diffusion coefficient of the sample was calculated by averaging values from all time intervals.

## Figures and Tables

**Table 1:** Summary of results from amplitude sweep tests of agarose–collagen hydrogels.

Sample Composition		LVER End			Average Moduli in LVER						Crossover Point					
Agarose	Collagen	Strain			G′			G″			G′			Strain		
(wt. %)	(wt. %)	(%)			(Pa)			(Pa)			(Pa)			(%)		
0.5	×	4.14	±	0.56	2488	±	146	217	±	27	358	±	12	88	±	12
	0.01	5.11	±	0.01	1184	±	30	70	±	2	134	±	14	204	±	8
	0.05	4.14	±	1.38	1265	±	110	118	±	14	103	±	13	228	±	6
	0.1	4.14	±	1.37	3122	±	679	294	±	67	336	±	284	85	±	49
1	×	2.86	±	0.30	7934	±	675	720	±	63	1097	±	403	68	±	4
	0.01	2.57	±	0.86	6270	±	149	549	±	42	890	±	277	107	±	38
	0.05	1.96	±	0.00	6514	±	543	570	±	82	606	±	96	89	±	22
	0.1	1.96	±	0.00	18,462	±	3187	1782	±	351	2102	±	1167	62	±	1
2	×	1.46	±	0.30	42,422	±	3549	3650	±	261	5423	±	1520	40	±	8
	0.01	0.75	±	0.00	25,225	±	808	2132	±	95	3850	±	604	37	±	1
	0.05	1.97	±	0.01	33,719	±	274	2972	±	141	5904	±	686	32	±	4
	0.1	1.36	±	0.85	35,844	±	639	3770	±	202	3829	±	623	85	±	25

## Data Availability

The data used in this study are available on request from the corresponding author.

## Supplementary Materials

The following supporting information can be downloaded at https://www.mdpi.com/article/10.3390/gels11060396/s1, Figure S1: Amplitude sweep (A) and frequency sweep (B) tests of 0.5 wt. % agarose hydrogels (reference and with each addition of collagen). Circles represent storage moduli G′ and triangles represent loss moduli G″; Figure S2: Amplitude sweep (A) and frequency sweep (B) tests of 1.0 wt. % agarose hydrogels (reference and with each addition of collagen). Circles represent storage moduli G′ and triangles represent loss moduli G″; Figure S3: Amplitude sweep (A) and frequency sweep (B) tests of 2.0 wt. % agarose hydrogels (reference and with each addition of collagen). Circles represent storage moduli G′ and triangles represent loss moduli G″; Table S1: Comparison of selected physicochemical properties of collagen and silk fibroin; Figure S4: Concentration profiles of eosin B in agarose–collagen hydrogels; Figure S5: Concentration profiles of methylene blue in agarose–collagen hydrogels; Table S2: Values of effective diffusion coefficient (D_eff_) and theoretical concentrations at the hydrogel interface (c_0_) of methylene blue and eosin B; Figure S6: Infrared spectrum (A), differential scanning calorimetry (B), and dynamical mechanical analysis (C) of collagen mass (VUP medical) used for sample preparation; Table S3: Parameters of rheology measurement of agarose–collagen hydrogels; Table S4: Structural formulas and molecular weights of methylene blue and eosin B; Table S5: Parameters of diffusion of model dyes in agarose–collagen hydrogels.
