# Supplementary material for: Impact of Collagen on the Rheological and Transport Properties of Agarose Hydrogels
Source: Gels. 2025 May 27;11(6):396. doi: 10.3390/gels11060396 (PMC12191895; doi:10.3390/gels11060396)
Supplement: Supplementary file 1 [file gels-11-00396-s001.zip › Supplementary materials.pdf]

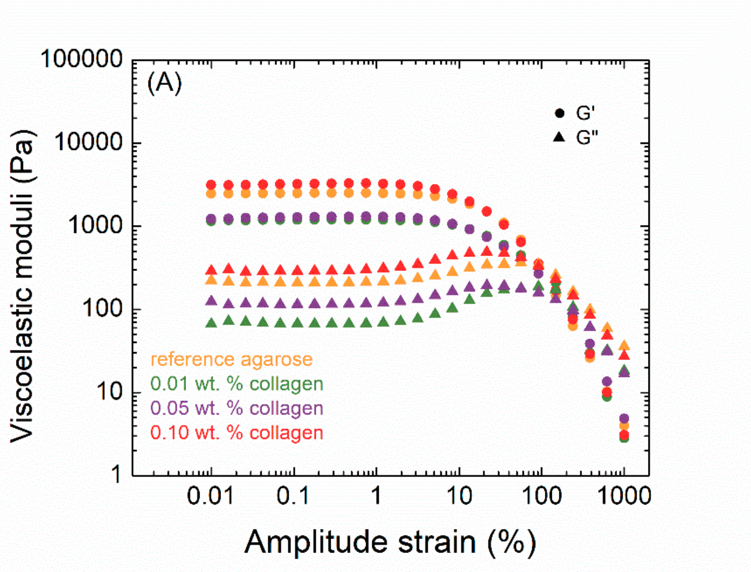

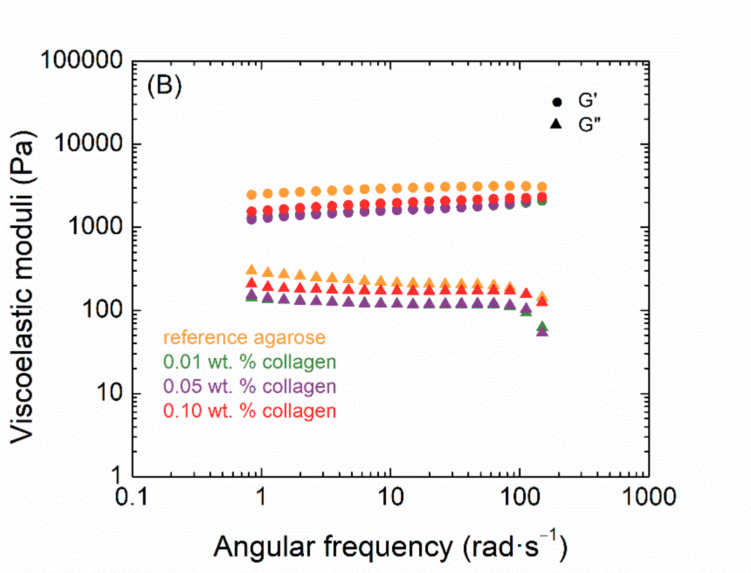

Figure S1. Amplitude sweep (A) and frequency sweep (B) tests of 0.5 wt. % agarose hydrogels (reference and with each addition of collagen). Circles represent storage moduli G' and triangles represent loss moduli G''.

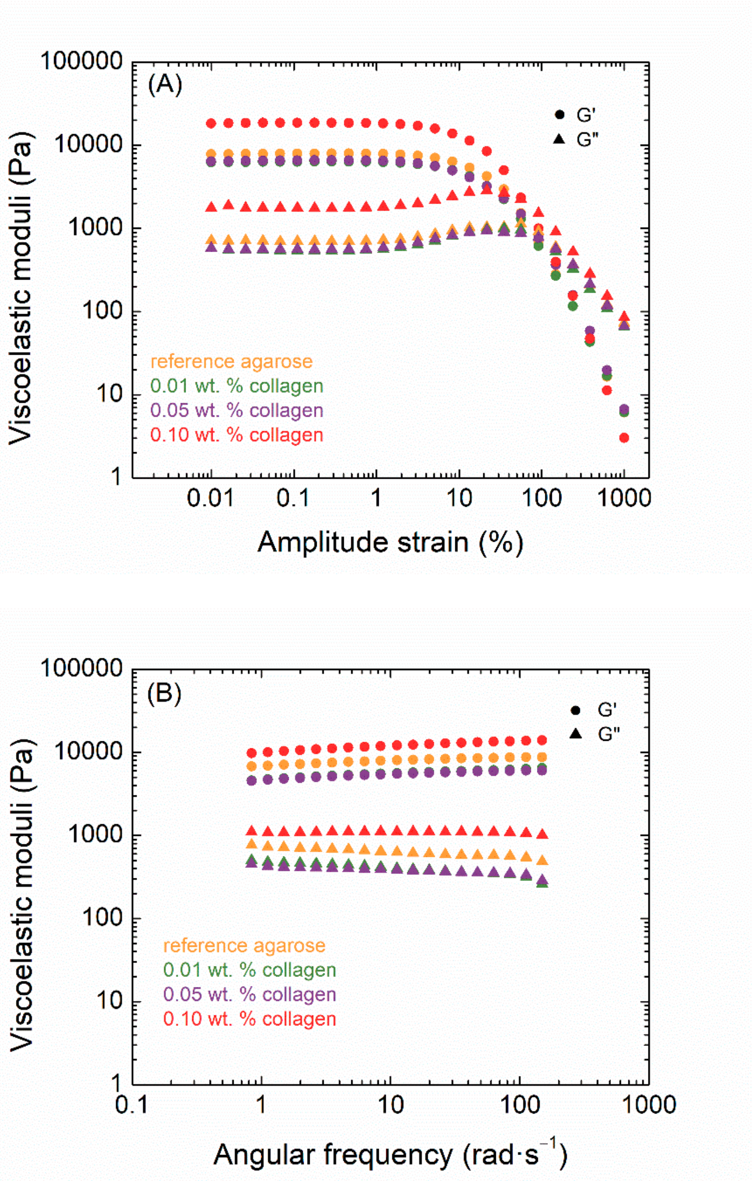

Figure S2. Amplitude sweep (A) and frequency sweep (B) tests of 1.0 wt. % agarose hydrogels (reference and with each addition of collagen). Circles represent storage moduli G' and triangles represent loss moduli G''.

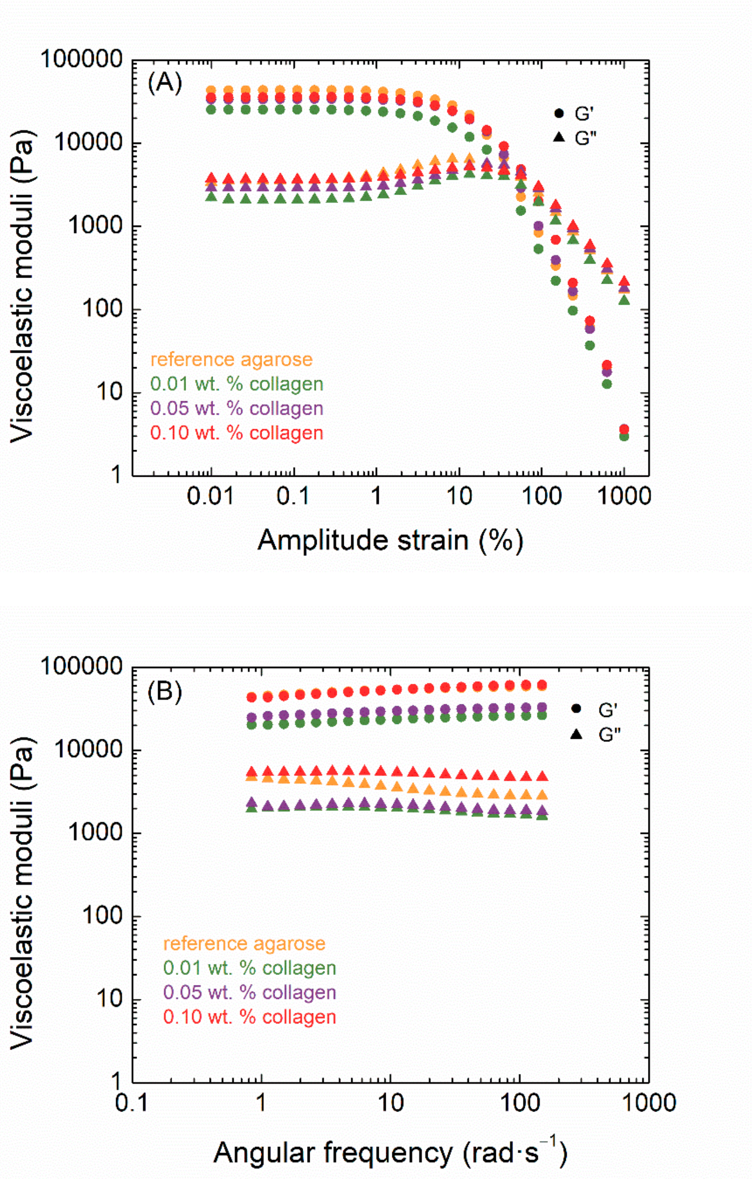

Figure S3. Amplitude sweep (A) and frequency sweep (B) tests of 2.0 wt. % agarose hydrogels (reference and with each addition of collagen). Circles represent storage moduli G’ and triangles represent loss moduli G’’.

Table S1. Comparison of selected physicochemical properties of collagen and silk fibroin.

Property	Collagen	Silk Fibroin
Origin	mammals, fish	Bombyx mori
Major amino acids	Gly, Pro, Hyp	Gly, Ala, Ser
Structure (secondary)	triple helix	β-sheet, α-helix
State in samples	dispersed fibrils in aqueous solution	dissolved polymer chains
Average molecular weight	300 kDa (tropocollagen)	200-400 kDa (heavy chain)
Isoelectric point (pI)	5.5-6.4	2.6-4.2
Charge at pH 7.4	negative	slightly negative

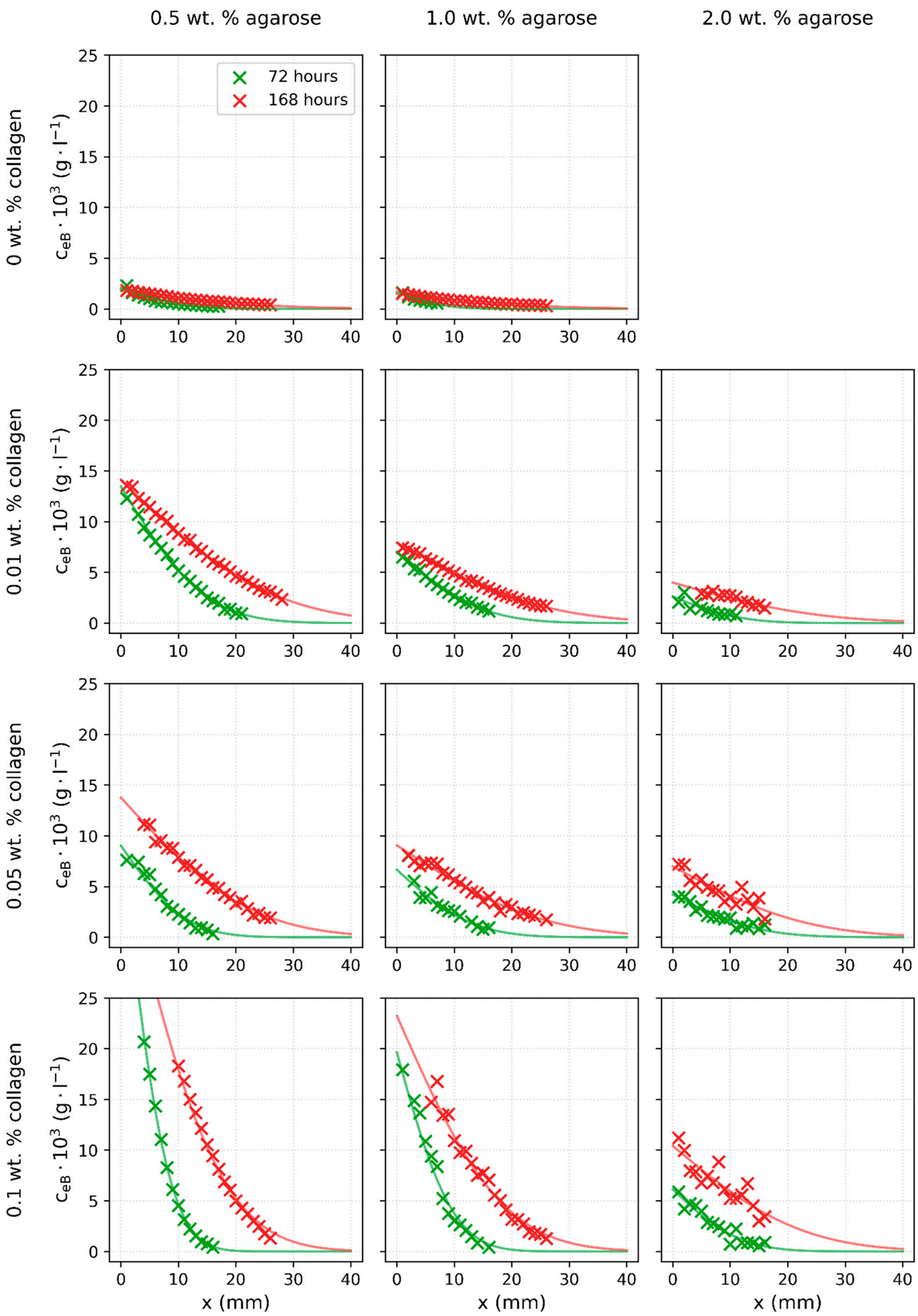

Figure S4. Concentration profiles of eosin B in agarose-collagen hydrogels.

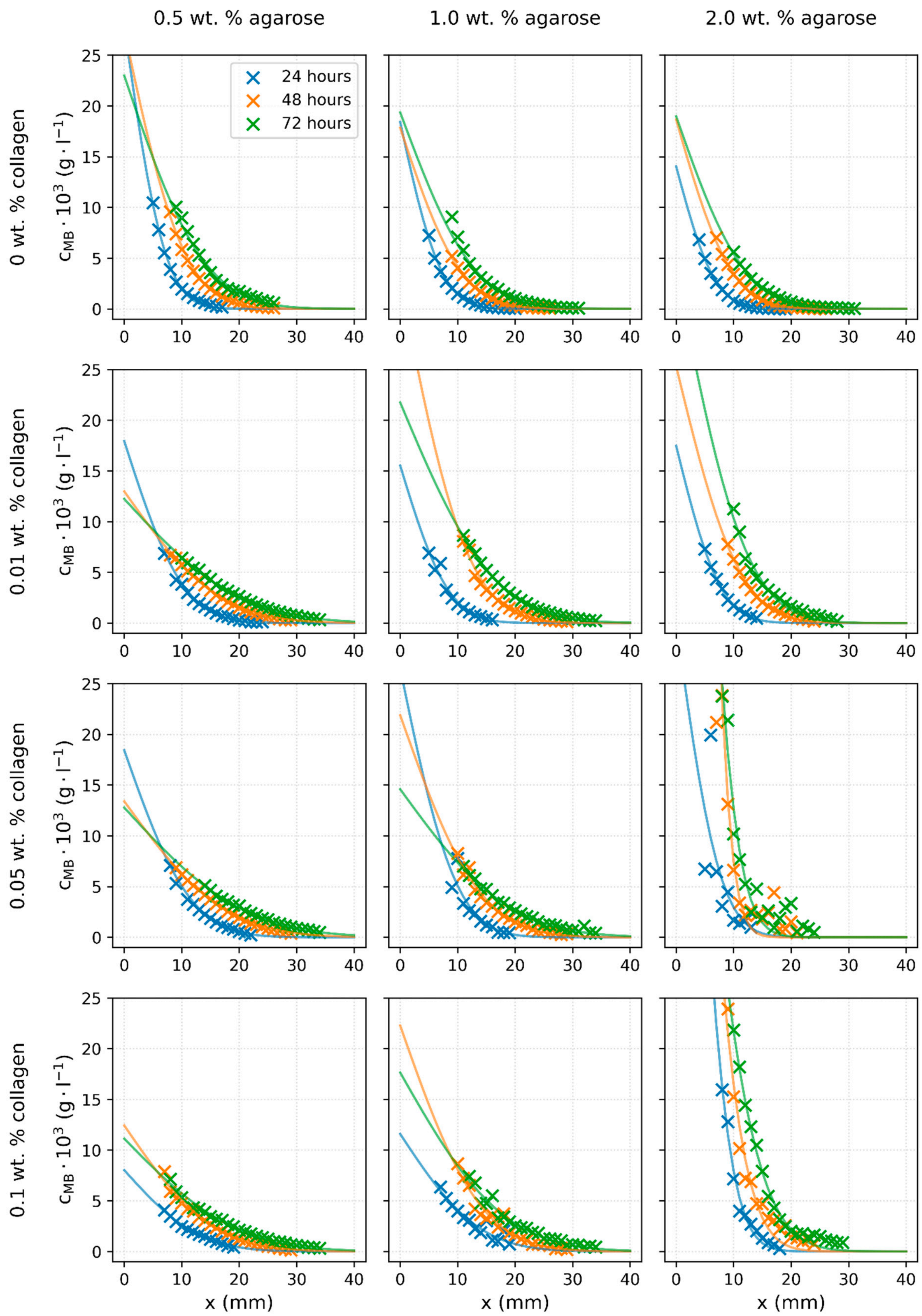

Figure S5. Concentration profiles of methylene blue in agarose-collagen hydrogels.

Table S2. Values of effective diffusion coefficients (D_{eff}) and theoretical concentrations at the hydrogel interface (c_0) of methylene blue and eosin B.

Sample composition		*Diffusion parameters - methylene blue*		*Diffusion parameters - eosin B*	
**Agarose** (wt. %)	**Collagen** (wt. %)	**$D_{eff} \cdot 10^{11}$** ($m^2 \cdot s^{-1}$)	**$c_0 \cdot 10^3$** ($g \cdot l^{-1}$)	**$D_{eff} \cdot 10^{11}$** ($m^2 \cdot s^{-1}$)	**$c_0 \cdot 10^3$** ($g \cdot l^{-1}$)
0.5	×	20.77 ± 1.28	30.00 ± 2.96	20.06 ± 5.19	1.97 ± 0.07
	0.01	45.11 ± 2.85	14.38 ± 0.18	30.14 ± 1.30	13.80 ± 0.33
	0.05	51.76 ± 1.59	14.87 ± 0.18	20.17 ± 2.30	11.39 ± 2.37
	0.1	49.21 ± 4.68	10.51 ± 0.13	10.96 ± 1.65	39.79 ± 0.22
1	×	19.27 ± 0.80	29.06 ± 3.05	18.10 ± 6.52	1.49 ± 0.09
	0.01	27.44 ± 1.84	23.66 ± 0.53	29.43 ± 0.67	7.44 ± 0.47
	0.05	37.11 ± 2.46	21.00 ± 0.35	25.86 ± 1.95	7.86 ± 1.20
	0.1	46.80 ± 8.31	17.16 ± 0.31	14.04 ± 1.10	21.44 ± 1.80
2	×	15.96 ± 0.25	22.52 ± 2.70	× ± ×	× ± ×
	0.01	20.21 ± 1.09	25.95 ± 0.51	23.83 ± 4.08	3.30 ± 0.69
	0.05	10.81 ± 4.04	73.67 ± 3.92	26.60 ± 1.16	5.60 ± 1.39
	0.1	15.97 ± 1.12	98.98 ± 0.93	20.64 ± 1.80	8.26 ± 2.15

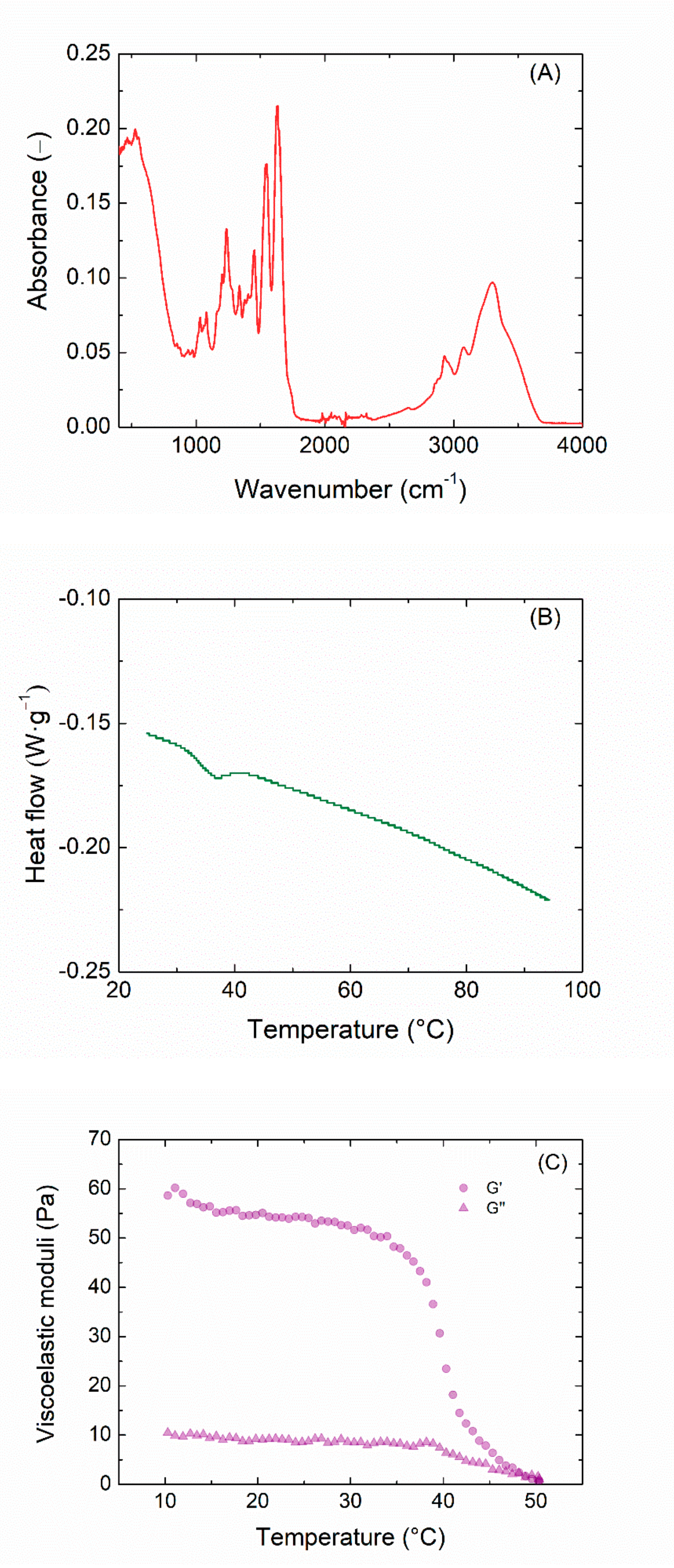

Figure S6. Infrared spectrum (A), differential scanning calorimetry (B), and dynamical mechanical analysis (C) of collagen mass (VUP medical) used for sample preparation.

Table S3. Parameters of rheology measurement of agarose-collagen hydrogels.

Parameter	***Amplitude sweep***	***Frequency sweep***
Conditioning step	300 s	300 s
Measuring gap	1000 μm	1000 μm
Temperature	25 °C	25 °C
Strain	0.01-1000 %	0.1 %
Frequency	1 Hz	0.01-100 Hz

Table S4. Structural formulas and molecular weights of methylene blue and eosin-B.

Methylene blue	*Eosin B*
M = 319.85 g·mol^{-1}	M = 580.09 g·mol^{-1}
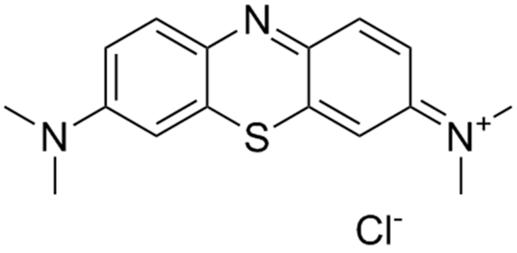	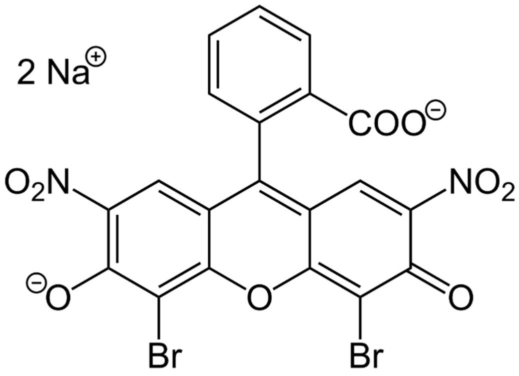

Table S5. Parameters of diffusion of model dyes in agarose-collagen hydrogels.

Model dye	***Eosin B***	***Methylene blue***
Concentration	0.05 $g \cdot dm^{-3}$	0.01 $g \cdot dm^{-3}$
Diffusion time	72 and 196 hours	24, 48, and 72 hours
Spectral range	300–700 nm	400–800 nm
Absorption maximum	520 nm	666 nm
